# The Parasitic Intracellular Lifestyle of Trypanosomatids: Parasitophorous Vacuole Development and Survival

**DOI:** 10.3389/fcell.2020.00396

**Published:** 2020-06-10

**Authors:** Marina Ferreira Batista, Carlos Alcides Nájera, Isabela Meneghelli, Diana Bahia

**Affiliations:** Departamento de Genética, Ecologia e Evolução, Instituto de Ciências Biológicas, Universidade Federal de Minas Gerais, Belo Horizonte, Brazil

**Keywords:** *Trypanosoma cruzi*, *Leishmania*, vacuole, parasitophorous vacuole, intracellular pathogen

## Abstract

The trypanosomatid (protozoan) parasites *Trypanosoma cruzi* and *Leishmania* spp. are causative agents of Chagas disease and *Leishmaniasis*, respectively. They display high morphological plasticity, are capable of developing in both invertebrate and vertebrate hosts, and are the only trypanosomatids that can survive and multiply inside mammalian host cells. During internalization by host cells, these parasites are lodged in “parasitophorous vacuoles” (PVs) comprised of host cell endolysosomal system components. PVs effectively shelter parasites within the host cell. PV development and maturation (acidification, acquisition of membrane markers, and/or volumetric expansion) precede parasite escape from the vacuole and ultimately from the host cell, which are key determinants of infective burden and persistence. PV biogenesis varies, depending on trypanosomatid species, in terms of morphology (e.g., size), biochemical composition, and parasite-mediated processes that coopt host cell machinery. PVs play essential roles in the intracellular development (i.e., morphological differentiation and/or multiplication) of *T. cruzi* and *Leishmania* spp. They are of great research interest as potential gateways for drug delivery systems and other therapeutic strategies for suppression of parasite multiplication and control of the large spectrum of diseases caused by these trypanosomatids. This mini-review focuses on mechanisms of PV biogenesis, and processes whereby PVs of *T. cruzi* and *Leishmania* spp. promote parasite persistence within and dissemination among mammalian host cells.

## Introduction

*Trypanosoma cruzi* and *Leishmania* spp. are evolutionarily closely related trypanosomatid protozoan parasites and the causative agents of Chagas disease and leishmaniasis, respectively, (Chagas, 1909; Brener, 1997; Harmer et al., 2018). These are classified by WHO as neglected tropical diseases (NTDs), and collectively affect over 20 million people worldwide – mainly populations living in remote, poorly developed areas (WHO, 2020a,b). *T. cruzi* and *Leishmania* spp. have complex and distinctive life cycles, and both are transmitted by insect vectors (triatomine bugs and phlebotomine flies, respectively) to various mammalian species (including humans) that act as persistent hosts (Ashford, 2000; Zingales et al., 2012; Kaufer et al., 2017). Trypanosomatids display remarkable plasticity in adapting to distinctive host organisms and environments, and in adapting and developing resistance to the action of drugs intended to control infection, thus presenting a challenge to any therapeutic strategy (Genois et al., 2014; Reis-Cunha et al., 2015; Laffitte et al., 2016; Reis-Cunha et al., 2018; Kaufer et al., 2019). Such adaptability is based on morphological and biochemical changes. For example, *T. cruzi* has five morphologically distinct developmental forms: non-infectious multiplicative epimastigote and infectious less-replicative metacyclic trypomastigote forms that colonize the insect vector, an intracellular amastigote form that multiplies within mammalian host cells, and infectious extracellular amastigote and trypomastigote forms that enter the mammalian host bloodstream (Lima et al., 2010; Ferreira et al., 2012).

*Leishmania* spp. have two clearly defined developmental forms: promastigote forms that colonize insect vectors (divided into non-infectious procyclic and infectious metacyclic subgroups), and amastigote forms that multiply within mammalian host cells (primarily macrophages; Tomlinson et al., 1995; Kaufer et al., 2017). The host cell interior is thus an important developmental environment for trypanosomatids, and they have developed various strategies for entering host cells. For this purpose, *T. cruzi* can utilize “passive” pathways such as endocytosis (Mortara et al., 2005, 2008; de Souza et al., 2010; Fernandes et al., 2015), or “active” parasite-mediated invasive pathways such as actin depolymerization induced in either phagocytic or non-phagocytic cells by extracellular amastigotes (Mortara, 1991; Mortara et al., 2008; Caradonna and Burleigh, 2011; Fernandes et al., 2013, 2015) or induction of lysosome-mediated membrane repair that promotes internalization (Fernandes et al., 2011).

In contrast, entry of *Leishmania* spp. into host cells occurs mainly via endocytosis, primarily by “professional phagocytes” such as macrophages and neutrophils. Neutrophils act as “Trojan horses,” they deliver parasites into host cells after being phagocytosed by macrophages (Farah et al., 1975; Chang and Dwyer, 1976; Ribeiro-Gomes et al., 2004). In addition, *Leishmania amazonensis* was recently reported to enter non-phagocytic cells (e.g., fibroblasts) using a Ca^2+^-dependent mechanism similar to one utilized by *T. cruzi*, involving membrane damage and repair via lysosomal exocytosis (Fernandes et al., 2015; Cavalcante-Costa et al., 2019).

Following cell invasion, intracellular obligate parasites use various strategies to withstand host cell immune responses and persist in the host cell. One such strategy, commonly observed in medically relevant infectious diseases, is formation of pathogen-containing vacuoles within host cells after pathogen internalization. Among trypanosomatids, only *T. cruzi* and *Leishmania* spp. are intracellular parasites of mammals.

After entering host cells, they are sheltered within a type of vacuole termed “parasitophorous vacuoles” (PVs), an essential preliminary step in further intracellular development of the parasites. PVs act as filters for nutrients to the detriment of host cell immune response factors, creating a niche for parasite differentiation, and/or multiplication. PVs also function as intermediary chambers facilitating development of parasites until they reach the cell cytoplasm, where they multiply and then exit the host cell (the case for *T. cruzi*; Basu and Ray, 2005; Barrias et al., 2013 for reviews), or until they are transferred safely from cell to cell without contacting the extracellular milieu (the case for *L. amazonensis*; Real et al., 2014). This mini-review focuses on mechanisms of PV biogenesis, and processes whereby PVs of *T. cruzi* and *Leishmania* spp. promote parasite persistence within and dissemination among mammalian host cells.

## Parasitophorous Vacuole Development in Trypanosomatids is an Evolutionary Adaptation for Intracellular Parasitism

Trypanosomatids, in their evolutionary history, were initially non-parasitic free-living organisms, as evidenced by their common ancestor *Bodo saltans*, and subsequently underwent selective pressure leading to development of the capacity to colonize host organisms, as either extracellular or intracellular parasites (Jaskowska et al., 2015). Ability to live inside host cells was a key evolutionary adaptation toward parasitism in mammalian hosts. For example, *T. brucei* (which is more closely related to *T. cruzi* than to *Leishmania*) does not require an intracellular/vacuolar environment in order to parasitize mammals, and exists exclusively as an extracellular parasite in the bloodstream of its mammalian host during its life cycle (Stevens et al., 1998; Hamilton et al., 2007). Adaptation to such an intracellular lifestyle has apparently involved genome reduction during the evolutionary history of many parasitic microorganisms (Casadevall, 2008). Genomes of the PV-forming intracellular parasites *Leishmania* spp. and *T. cruzi* are smaller than those of extracellular parasites such as *T. brucei*, indicating that PV biogenesis was a later adaptation that was beneficial to previously exclusively extracellular parasites (Jaskowska et al., 2015). *T. cruzi* likely appeared earlier than *Leishmania* spp. during trypanosomatid evolutionary history, which could account for the persistence of *T. cruzi* in mammalian host organisms as both extracellular (like *T. brucei*) and intracellular forms (like *Leishmania* spp.). From an evolutionary point of view, PV biogenesis may be a refined adaptation for parasitism that enhanced the fitness of trypanosomatid parasites involved in medically relevant pathologies.

Although both *T. cruzi* and *Leishmania* spp. depend on PV development to persist inside host cells, the former is sheltered transiently by PVs and then is released to host cell cytosol where it multiplies, whereas the latter is sheltered permanently by PVs throughout its intracellular life cycle, and multiplies within PVs (Fernandes et al., 2011; Fernandes and Andrews, 2012). Its release from these PVs to cytoplasm depends on an acid pH-dependent signaling event promoted by lysosome recruitment toward PV; this leads to parasite exit into cytosol, where they differentiate into replicative amastigote forms and multiply (Ley et al., 1990; Andrade and Andrews, 2004; Fernandes and Andrews, 2012). *Leishmania* spp., on the other hand, take advantage of PVs to differentiate into amastigotes and also to multiply. Analogously to the diverse spectrum of disease outcomes in leishmaniasis (Burza et al., 2018), PVs of *Leishmania* spp. display diverse morphologies depending on species: the great majority of *Leishmania* spp. (including *L. major* and *L. donovani*) develop in single compact PVs, whereas members of the *L. mexicana* complex (*L. amazonensis*, *L. mexicana*) multiply within larger PVs (Real and Mortara, 2012).

Thus, PVs are customized to fulfill the requirements of parasites for their intracellular development, as reflected by the construction of doubly infected, chimeric PVs, i.e., single pathogen-containing vacuoles that host different parasite species simultaneously. In a model system of chimeric vacuoles hosting *L. amazonensis* amastigotes (primo-infection) and *L. major* promastigotes (superinfection), the latter was unable to differentiate into amastigote form (Real et al., 2010). In another model of chimeric vacuoles using *L. amazonensis* large PVs as recipient vacuoles for *T. cruzi*, the latter differentiated into replication-competent amastigote forms not in cytosol but within phagolysosome-like *L. amazonensis* large PVs, indicating that trypomastigote-to-amastigote differentiation of *T. cruzi* occurs under the acidic pH of PVs and precedes the release from PV to cytosol (Pessoa et al., 2016). This finding is consistent with previous reports that PV alkalinization impairs parasite PV escape (Ley et al., 1990; Stecconi-Silva et al., 2003). Rather than being released from PVs (as *T. cruzi* does), *Leishmania* spp. remain associated with PVs even during cell-to-cell parasite spreading. *L. amazonensis* takes advantage of host macrophage apoptosis to transfer from macrophage to macrophage *in vitro*, and remains associated with host lysosomal components on its surface that trigger anti-inflammatory cytokine production by recipient non-apoptotic macrophages (Real et al., 2014). PVs thus provide an additional shelter from the extracellular milieu and immune system surveillance, and participate in the late intracellular life cycle of parasites; i.e., egress from host cells and reinfection of new ones.

## Parasitophorous Vacuolar Biogenesis and Maintenance Depend on Host Cell Machinery

Several parasite species utilize a strategy based on formation of a specifically designed, customized PV during the process of cell infection. Intracellular persistence of trypanosomatids depends on several host-related features. In contrast to various intracellular pathogens that interfere with phagosome maturation to avoid transport to lysosomes, *Leishmania* spp. and *T. cruzi* recruit lysosome markers during the process of infection, and need an acidic environment to maintain their intracellular life cycle. This strategy requires that the parasites remodel and subvert the host endolysosomal pathway to benefit themselves.

In cells infected with *L. amazonensis*, an exchange of biomolecules (e.g., lipids, proteins, and sialoglycoproteins) between cells and parasites was observed following contact. The PV was labeled with the same molecular markers of the parasite, indicating that in addition to host cell internalized components, there is a shedding of proteins from the intracellular parasites to PV (Henriques and De Souza, 2000). Henriques et al. (2003) confirmed the transfer of lipids by labeling macrophages with ^32^P and then exposing the cells to *L. amazonensis*. The main phospholipid component of PV was phosphatidylcholine. Changes were observed in PV protein composition in relation to time of infection and morphological form of the parasite (Henriques et al., 2003).

Gagnon et al. (2002) showed, based on observation of calnexin markers, that endoplasmic reticulum (ER) participates in formation of phagosomes in macrophages, by fusion with plasmatic membrane during early phagocytosis and its subsequent maturation. Entry of pathogens such as *L. donovani* into macrophages evidently required ER proteins such as calnexin and calreticulin, indicating participation of ER in the internalization process. Similarly, Canton et al. (2012) showed that ER elements are involved in PV formation in *L. amazonensis*, through action of SNARE protein.

*Leishmania donovani* also is able to upregulate Rab5a, an early endosome protein. The parasite retains Rab5a, along with its effector protein EEA1, in PVs, thereby forming and maintaining an early endosome compartment and delaying maturation (Verma et al., 2017). Such delay is observed in many other *Leishmania* species and is mediated by parasite surface components such as lipophosphoglycan (LPG; Lodge and Descoteaux, 2005), favoring differentiation into amastigote forms of promastigotes otherwise sensitive to the harsh environment of fully matured phagolysosomal vacuoles. In contrast, *L. amazonensis* acquires Rab5a and EEA1 soon after internalization but does not maintain these early endosome markers; rather, it rapidly acquires late endosome and lysosome markers (Courret et al., 2002; reviewed by Veras et al., 2019). For *L. amazonensis* (and possibly other members of the *L. mexicana* complex), PV maturation in terms of acquisition of lysosomal membrane markers and content is accompanied by a striking increase in PV volumetric size, which is dependent on host cell factors such as lysosomal traffic regulator LYST/Beige (Wilson et al., 2008), CD36 receptor (Okuda et al., 2016), and V-ATPase subunit d isoform 2 (ATP6V0d2; Pessoa et al., 2019). Lysosome marker recruitment and PV enlargement are impaired when the host cell lacks CD36 receptor, thereby impairing parasite multiplication as well (Okuda et al., 2016). In another mechanism possibly involving CD36, ATP6V0d2 knockdown depletes macrophage cholesterol and inhibits PV enlargement without impairing parasite multiplication (Pessoa et al., 2019). Increase of cholesterol level by addition of oxidized low-density lipoprotein (ox-LDL), of which CD36 is the receptor, results in PV enlargement and impaired parasite multiplication.

Initial parasite-host cell interaction leading to parasite internalization involves recognition of conserved parasite components, termed pathogen-associated molecular patterns (PAMPs), by host pattern recognition receptors (PRRs); the parasites then take advantage of adhesion to host cells to access safe intracellular environments (Bahia et al., 2018). Such interaction modifies cellular signaling pathways and thereby determines parasite fate. Activation of host signaling pathways [phosphatidylinositol-3-kinase/protein kinase C (PI3K/PKC) – mTOR pathway and endolysosomal pathway for *T. cruzi*; phagocytic pathway for *Leishmania* spp.] leads to parasite internalization and PV formation (Romano et al., 2009; Martins et al., 2011; Salassa and Romano, 2018; Ferreira et al., 2019).

*Trypanosoma cruzi* endocytic entry into non-professional phagocytic cells has been clearly shown to require lysosome recruitment (Meirelles et al., 1987; Carvalho and de Souza, 1989) and actin reorganization (Rosestolato et al., 2002). Metacyclic and culture-derived trypomastigote forms both depend on transient presence of Ca^2+^ during entry into cells driven by parasite surface molecules (gp82- MT/oligopeptidase B-CDT; Yoshida, 2006). Intact microtubule machinery is essential for *T. cruzi* internalization (Rodriguez et al., 1996; Rosestolato et al., 2002). During *T. cruzi* infection, microtubules play a role in directing lysosomes to PVs, which act as sites of microtubule organization (Tyler et al., 2005).

PV establishment in *T. cruzi* is directly related to its mechanism of entry into cells. Internalization of *T. cruzi* in non-phagocytic cells clearly depends on early lysosomal exocytosis to parasite infection sites (Tardieux et al., 1992). Following parasite-induced plasma membrane damage, membrane repair is stimulated via lysosomal exocytosis, and *T. cruzi* takes advantage of this process to enter the cell (Fernandes et al., 2011). Woolsey et al. (2003) demonstrated that trypomastigotes can also utilize a different invasion route which is dependent on phosphatidylinositol 3-phosphate (PIP-3) plasma membrane-associated molecules in non-phagocytic cells. Only 20% of the analyzed parasite population presented early endosomal markers (e.g., EEA1), in contrast to the 50% of the population that invaded via class I phosphatidylinositol-3-kinases (PI3K)-mediated PI3-P accumulation. These findings indicate that the main endocytic internalization pathway of *T. cruzi* in non-professional phagocytic cells does not require lysosomal exocytosis, contrary to previously proposed mechanisms.

Parasitophorous vacuole features formed during these two processes are distinctive. In the lysosome-dependent pathway (early lysosome fusion), Ca^2+^-dependent exocytosis of lysosomes is activated and lysosomes fuse with nascent PVs (Tardieux et al., 1992; Fernandes et al., 2011). In contrast, in the lysosome-independent pathway (late lysosome fusion), parasite entry occurs through membrane invagination resulting from PIP3 accumulation, and lysosome markers are acquired only during PV maturation (Woolsey et al., 2003).

In HeLa cells, extracellular amastigotes induce PI3K pathway to promote rearrangement of actin cytoskeleton and their own phagocytosis (Ferreira et al., 2019). This process likely interferes with PV formation, although the mechanism remains unclear.

**FIGURE 1:**
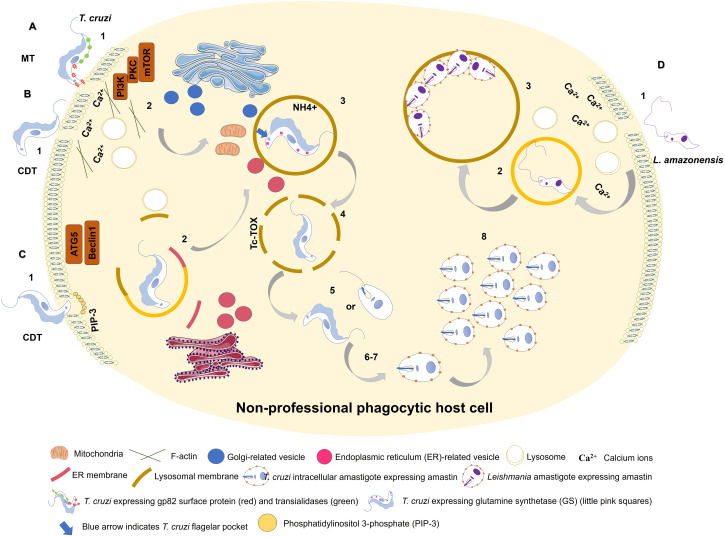
Entry of *Trypanosoma cruzi* metacyclic trypomastigote (MT) forms into non-professional phagocytic cell **(A)**. 1: MT entry requires parasite surface protein gp82 and recruitment of lysosomes to the infection site in a Ca^2+^-dependent manner. 2: MT invasion activates PI3K/PKC-mTOR signaling and F-actin disruption. 3: Acidic parasitophorous vacuole is formed as *T. cruzi* begins internalization. Mitochondria are localized near flagellar pocket (blue arrow). *T. cruzi* vacuolar closure and maturation/acidification involve continuous communication with ER/Golgi vesicles. Glutamine synthetase (GS) controls pH by regulating NH4^+^ vacuolar content (small pink squares). 4: *T. cruzi* vacuolar degradation. 5: *T. cruzi* vacuolar escape into cytoplasm. MT form remains intact, or an intermediate form between MT and intracellular amastigote (IA) is present. 6: *T. cruzi* extravacuolar differentiation into IA forms. 7: Free *T. cruzi* IA in cytoplasm. 8: *T. cruzi* IA cytoplasmic replication. *T. cruzi* culture-derived trypomastigote (CDT) invasion by lysosome-dependent pathway (early lysosome fusion; **B)**. 1: CDT induces membrane damage. 2: CDT requires parasite oligopeptidase B surface protein and recruitment of lysosomes to the infection site in a Ca^2+^-dependent manner. F-actin disruption also plays a role in CDT invasion. 3-8: Similar to **(A)**. *T. cruzi* culture-derived trypomastigote (CDT) invasion by lysosome-independent pathway (late lysosome fusion; **C)**. 1: CDT invades by membrane invagination resulting in a PI3K-dependent PIP3 accumulation. In deprivation of nutrients host cell increases CDT internalization through ATG5/Beclin1 pathway. 2: lysosome markers are acquired only during PV maturation. ER membrane is donated to PV during its membrane construction. 3-8: Similar to (A). Model of *L. amazonensis* non-phagocytic cell internalization as proposed by Cavalcante-Costa et al. (2019); **D)**. 1: *L. amazonensis* induces membrane damage and Ca^2+^ recruitment during invasion process. 2: *L. amazonensis* vacuolar formation and maturation. 3: *L. amazonensis* multiplication into large vacuoles.

Martins et al. (2011) showed that both metacyclic and culture-derived trypomastigotes elicit lysosome recruitment during invasion by activating autophagy-related signaling pathways. Parasite surface glycoprotein gp82 and host actin remodeling are required for induction of lysosome recruitment to plasma membrane during metacyclic invasion through activation of PI3K/PKC-mTOR pathway (Martins et al., 2011). In contrast, invasion of culture-derived trypomastigotes depends on activation of autophagy-related proteins ATG5 and Beclin, independently of mTOR pathway (Romano et al., 2009; Figure 1).

Host autophagy processes, in addition to regulating invasion, have been shown to affect PV maturation (Ghartey-Kwansah et al., 2020). In *L. donovani*, both canonical and non-canonical autophagy are triggered, at different infection time points (Pitale et al., 2019). Salassa and Romano (2018) suggested that autophagy is involved in PV maturation in *T. cruzi* infection, based on observed recruitment of LC3 (an autophagosome marker) to PV. Confirmation of this idea will require elucidation of the process involved. e.g., canonical autophagy, xenophagy, or LC3-associated phagocytosis (Salassa and Romano, 2018).

Intracellular parasites exploit host membrane resources and organelles to promote PV maintenance and maturation, in order to complete their life cycle. Reignault et al. (2019) observed formation of PVs during the first 2 h (not later times) of internalization by peritoneal macrophages of *T. cruzi* amastigotes and trypomastigotes. Electron microscopic and 3D reconstruction techniques indicated that during biogenesis of *T. cruzi* PVs, ER and lysosomes act as membrane donors for generation of PVs. Morphological changes were observed in cellular distribution of Golgi complex and mitochondria during PV biogenesis; these organelles moved from the perinuclear region to the PV vicinity. No membrane fusion with Golgi complex or mitochondria was observed; however, it is conceivable that both organelles function in synchrony with PV development, in view of their proximity. In the context of PV development, the observed exchange of membranes between parasite and PV suggest occurrence of emergent signaling pathways between parasite and host cell, and indicate involvement of the host actin cytoskeleton, which surrounds the PV from its biogenesis until its rupture (Reignault et al., 2014, 2019).

Lysosome-mediated PV acidification is necessary for both PV maturation and trypomastigote-to-amastigote differentiation; it enables proper functioning of parasite-derived Tc-TOX protein (de Souza et al., 2010). Carvalho and de Souza (1989) were the first to suggest a *T. cruzi* mechanism of PV dissolution. Stecconi-Silva et al. (2003) demonstrated later that Tc-TOX induces formation of pores to degrade PV membrane, thereby promoting parasite release into host cell cytoplasm.

## Trypanosomatid Strategies for Overcoming Host Cell Defenses

Intracellular parasites are able to persist and overcome host cell defense mechanisms through a variety of strategies, e.g., secreting proteins, hijacking host proteins, and recruiting host proteins/structures. Certain parasite-derived factors have been implicated in subversion of host cell functions.

Lipophosphoglycan, a glycoconjugated virulence factor on *Leishmania* spp. surfaces, enhances parasite survival by targeting host defense proteins. During *L. donovani* phagocytosis, LPG delays PV maturation by inducing F-actin depolymerization around the PV site, resulting in inhibition of vesicular trafficking holding (e.g., lack of LAMP-1 marker; Winberg et al., 2009), and of recruitment to PV of protein kinase Cα (PKCα), a kinase involved in F-actin degradation and regulation of PV development (Holm et al., 2001). GP63, another virulence factor, is a metalloprotease surface protein that interferes with PV acidification. da Silva Vieira et al. (2019) reported that GP63 and LPG expression varies in *L. braziliensis*, and that these PV effects are strain-specific (da Silva Vieira et al., 2019).

Certain host ER- and Golgi-localized N-ethylmaleimide-sensitive-factor attachment protein receptors (SNAREs) are related to endosome/lysosome fusion and are coopted by trypanosomatids for PV biogenesis (Ndjamen et al., 2010; Canton and Kima, 2012; Cueto et al., 2017). Vacuoles containing either *L. donovani* or *L. pifanoi* recruit endoplasmic markers such as calnexin and SNARE Sec22b to PV formation sites during phagocytosis (Ndjamen et al., 2010). *L. amazonensis* engages SNAREs (syntaxin-5, Sec22b) that play important roles in PV development; syntaxin-5 inhibition blocked PV enlargement and reduced parasite burden (Canton and Kima, 2012), and Sec22b and syntaxin-5 participated in trafficking of parasite-derived molecules in host cells (Canton and Kima, 2012; Arango Duque et al., 2019).

*Leishmania* spp. metacyclic promastigotes utilize the host classic exocytic pathway (ER/Golgi complex) to deliver GP63 and LPG from PVs toward the extracellular milieu, thereby affecting parasite persistence (Arango Duque et al., 2019). Casgrain et al. (2016) observed that *L. mexicana* GP63 cleaved VAMP3 and VAMP8, two endocytic SNAREs associated with phagosome biogenesis and function, and helped maintain parasite intracellular development to allow PV expansion. Matte et al. (2016) reported that cleavage of VAMP8 by *L. major* GP63 was associated with inhibition of LC3 recruitment to phagosomes during LC3-associated phagocytosis (LAP). This pathway is typically activated by external particles already contained within a single-membraned phagosome or endosome, and leads to deposition of LC3 on the cytosolic side of the phagosome, thereby promoting more rapid fusion with lysosomes (Evans et al., 2018). Disruption resulting from VAMP8 cleavage impairs host cell antimicrobial machinery (Matte et al., 2016).

**FIGURE 2:**
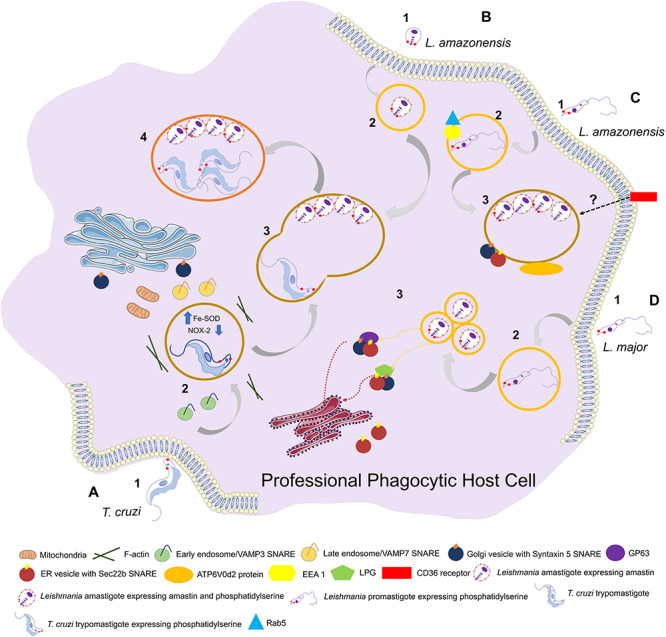
Entry of trypanosomatids in a professional phagocytic cell. **(A)** 1: Uptake of *Trypanosoma cruzi* trypomastigote by phagocytosis. This process can be facilitated through expression of phosphatidylserine (PS) in parasite surface. VAMP3 (SNARE) recruitment to entry site in early-phase infection. 2: *T. cruzi* vacuolar closure and maturation. VAMP7 (SNARE) is recruited and maintained during PV maturation. Parasite uses Fe-SOD to reduce the amount of O_2_^–^ produced by host (NOX-2) inside phagosome. PS exposition creates a permissive state to parasite survival. **(B)** 1: Uptake of *L. amazonensis* amastigote by phagocytosis. 2: *L. amazonensis* vacuolar formation and maturation. 3: *L. amazonensis* and *T. cruzi* vacuolar fusion. 4: Chimeric vacuole formation with *L. amazonensis* amastigotes and *T. cruzi* trypomastigotes. **(C)** 1: Uptake of *L. amazonensis* promastigote by phagocytosis. 2: *L. amazonensis* vacuolar formation and maturation. Rab5 and EEA1 endosome markers are acquired after internalization. 3: *L. amazonensis* multiplication into large vacuoles. ATP6V0d2 subunit of V-ATPase participates in cholesterol influx, an essential process in PV maintenance. Syntaxin 15 and Sec22b are SNAREs that play important roles in PV development. **(D)** 1: Uptake of *L. major* by phagocytosis. 2: *L. major* vacuolar formation and maturation. The parasite is developed in a single compact PV. 3: *L. major* multiplication into tight individual vacuoles. Parasite virulence factors GP63 and LPG are transferred to host cell (ER/ERGIC; “ER-Golgi intermediate compartment”) by SNAREs. Both promastigotes and amastigotes of *L. amazonesis* and *L. major* can present PS in its surface with the same functions as in *T. cruzi* facilitating parasite phagocytosis and, once the parasite is inside the cell, aiding in its survival.

In *T. cruzi*, two SNAREs (VAMP3, VAMP7) are recruited to PVs at different times during PV development: VAMP3 appears only in early phases following parasite internalization, whereas VAMP7 is readily recruited and maintained throughout PV maturation and maintenance, and is essential for parasite invasion and lysosome-PV fusion events (Cueto et al., 2017; Figure 2).

Amastin proteins, a family of stage-specific parasite surface factors first described in *T. cruzi* and showing upregulated expression in amastigote forms (Teixeira et al., 1994; Cruz et al., 2012), are reportedly involved in parasite intracellular survival and PV biogenesis (Cruz et al., 2012; de Paiva et al., 2015). The major amastin subfamily in *Leishmania* spp., termed δ-amastins, includes (~42 isoforms. Genome analysis of *L. amazonensis* showed that amastin subfamilies are species-specific, and show correlations with disease outcomes and PV volumetric size (Real et al., 2013). In *L. braziliensis*, δ-amastin knockdown reduced parasite PV attachment, thereby inhibiting multiplication and release into cytosol both *in vitro* and *in vivo* (Teixeira et al., 1994; de Paiva et al., 2015). *T. cruzi* has a smaller number of δ-amastin gene copies (12 in total) than do *Leishmania* spp. δ-amastin superexpression in *T. cruzi in vitro* was correlated with rapid differentiation of culture-derived trypomastigotes into intracellular amastigote forms, and with host cell egress. Overexpression of δ-amastin in *T. cruzi* extracellular amastigotes *in vivo* led to earlier parasite tropism (relative to wild-type) toward livers of infected mice (Cruz et al., 2012). Thus, parasite-derived membrane factors display a wide variety of functions depending on trypanosomatid species, ranging from parasite extracellular morphological development and parasite tropism in host organisms, to intracellular multiplication and PV biogenesis.

Another family of proteins, the trans-sialidases (TSs), also play important roles in parasite-PV interactions. Freire-de-Lima et al. (2015) showed that *T. cruzi* TSs help the parasite salvage host cell sialic acid for its own benefit. Besides their well-documented involvement in adhesion, invasion, and immune modulation, TSs facilitate parasite escape from PVs to cytosol, but the mechanism for this is unclear (Freire-de-Lima et al., 2015, 2017; da Fonseca et al., 2019). *T. cruzi* culture-derived trypomastigotes, in comparison with metacyclic forms, express higher TS levels and escape earlier from PVs throughout their intracellular life cycle, suggesting a link between TSs and PV escape. In infected cells lacking surface sialic acid and lysosome membranes, TS-overexpressing metacyclic forms and culture-derived trypomastigotes with high native TS expression show similar PV escape kinetics (Rubin-de-Celis et al., 2006, Rubin-de-Celis and Schenkman, 2012).

Crispim et al. (2018) showed that the ATP-dependent enzyme glutamine synthetase (GS) is associated with PV evasion in *T. cruzi*. GS regulates the level of metabolites derived from amino acid consumption by converting accumulated NH4^+^ and glutamate into glutamine. Blocking of GS by methionine sulfoximine (MS) inhibited trypomastigote PV escape into cytoplasm *in vitro*. *T. cruzi* PV escape is associated with acidic pH environment, and GS therefore may regulate intravacuolar NH4^+^ content and acidification (Crispim et al., 2018). Another enzyme associated with parasite survival within PVs is cytosolic iron superoxide dismutase (Fe-SOD), an O2^–^ catabolizing enzyme. In *T. cruzi*, Fe-SOD reduces the amount of O2^–^ produced by host NADPH oxidase (NOX-2) inside phagosomes, thereby counteracting host cell oxidative stress involved in defense against intracellular pathogens. Fe-SOD overexpression in parasites *in vivo* resulted in increased parasitemia and parasite burden in infected mice (Martínez et al., 2019).

Last but not least, recognition of phosphatidylserine (PS) on the membrane surface of apoptotic cells (apoptosis) is necessary for its elimination by phagocytes (endocytosis) without causing inflammation. A reproduction of this mechanism, termed “apoptotic mimicry,” is used by some intracellular parasites, including *T. cruzi* (trypomastigote forms) and *Leishmania* spp. (promastigote and amastigote forms), during the invasion process and maintenance of infection. In classical mimicry, the parasite expresses PS in order to be phagocytosed by macrophages, and a permissive state is created by decrease of NO production through induction of cytokines (e.g., TGF-β1; anti-inflammatory cytokine) and synthesis of IL-10, allowing the parasite to survive inside the host cell. In contrast, in non-classical mimicry the host cell expresses PS during the infection process. *Leishmania* spp., for example, initially colonizes a neutrophil and then induces its PS expression in order to be phagocytosed by a macrophage – the ideal host for the parasite. The amount of PS expressed determines the infection capacity of the parasite, and depends on an intrinsic pressure upon the host’s immune system (De Freitas Balanco et al., 2001; DaMatta et al., 2007; El-Hani et al., 2012; Wanderley et al., 2020).

## Conclusion

*Trypanosoma cruzi and Leishmania* spp. are trypanosomatid pathogens that depend on customized PV niches during the infection process. PV development mechanisms are complex, and vary among parasite species and strains. During evolution, mammalian host cells have adapted to resist invasion of intracellular parasites by establishment of hostile intracellular environments. Development of more efficient therapeutic strategies against Chagas disease and leishmaniasis will require better understanding of the PV processes related to such structural biogenesis, and the mechanisms whereby parasite factors subvert host cell responses.

## Author Contributions

MB, CN, IM, and DB conceived and wrote the manuscript.

## Conflict of Interest

The authors declare that the research was conducted in the absence of any commercial or financial relationships that could be construed as a potential conflict of interest.
